# Supplementary material for: Digital spatial profiling of the microenvironment of muscle invasive bladder cancer
Source: Commun Biol. 2024 Jun 18;7:737. doi: 10.1038/s42003-024-06426-9 (PMC11189454; doi:10.1038/s42003-024-06426-9)
Supplement: Supplementary file 2 — Supplementary Information [file 42003_2024_6426_MOESM2_ESM.pdf]

Variable		Number
**Treatment**	Radiotherapy	12
	Radiotherapy + CON	13
**Median age (range) years)**	76.6 (51.5 – 86.8)	
**Gender**	Male	21
	Female	2
**Stage**	T1	2
	T2	19
	T3	2
	T4	2
**Grade**	2	2
	3	23
**TURBT**	Biopsy	11
	Partial	7
	Complete	7
**Concurrent carcinoma in situ**	Absent	6
	Present	19
**Median Haemoglobin (range) g/dL**	14.60 (9.80, 17.10)	

Supplementary Table 1: Clinical and histological characteristics of patients in dataset from the BCON cohort. CON = carbogen plus nicotinamide; TURBT = Transurethral resection bladder tumour.

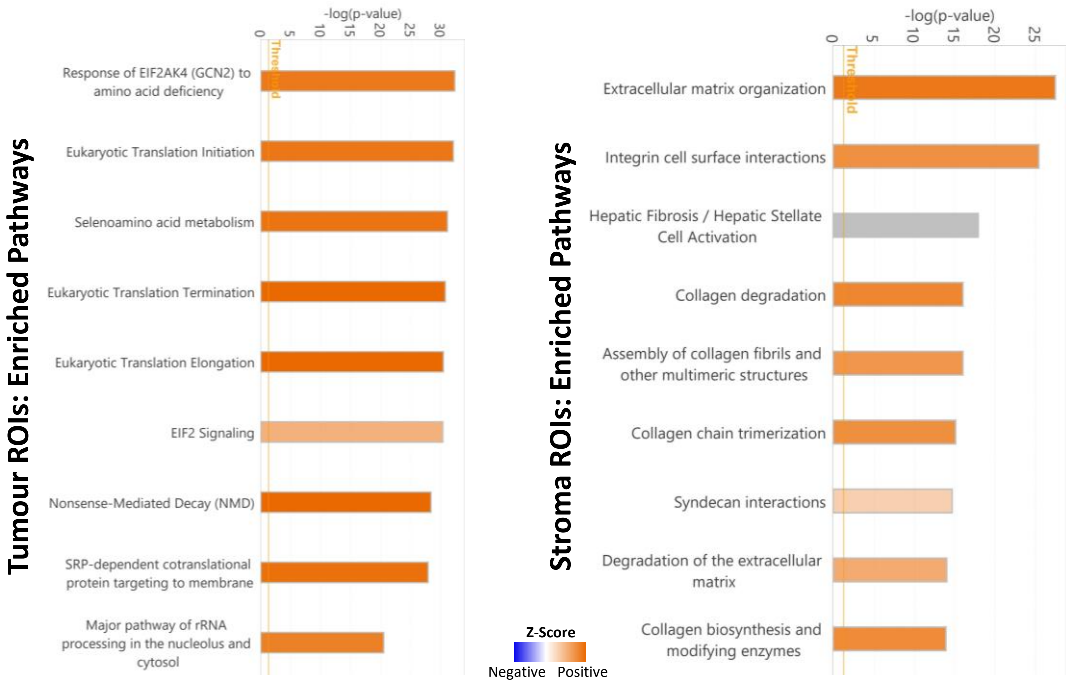

Supplementary Figure 1: IPA analysis of differentially expressed genes in (left) tumour and (right) stroma ROIs identified in figure 2C. Full results are available in supplementary tables. Tumour ROIs are enriched in EIF2 signalling and genes involved in translation elongation and initiation, while stroma ROIs are enriched in collagen and ECM related pathways.

Basal Tumour ROIs: Upstream Activators

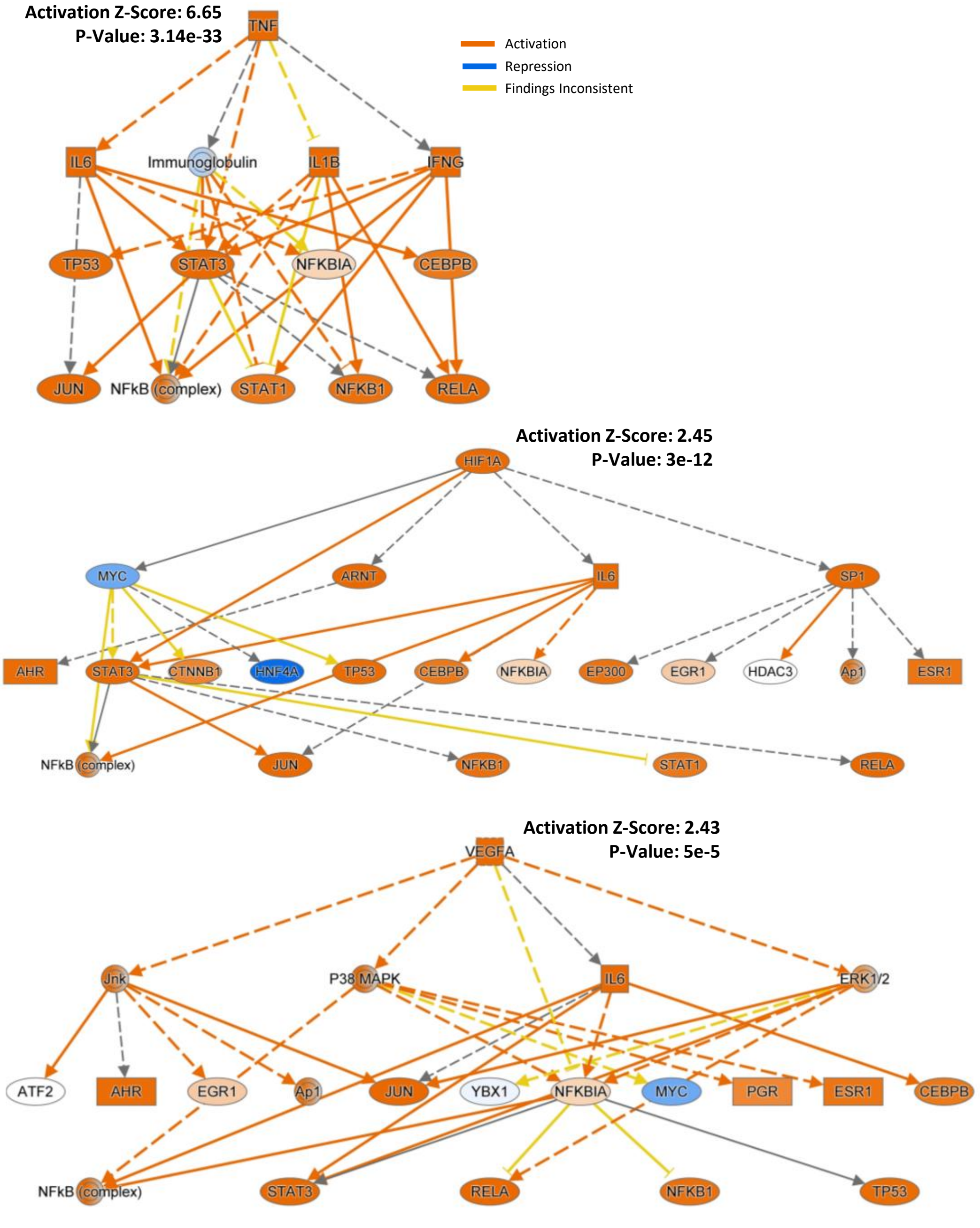

Supplementary Figure 2: Upstream activator analysis of basal tumour differentially expressed genes identified in Figure 3C. Genes enriched in basal tumours are consistent with activation of TNF, HIF1A and VEGFA. Full results can be found in supplementary tables.

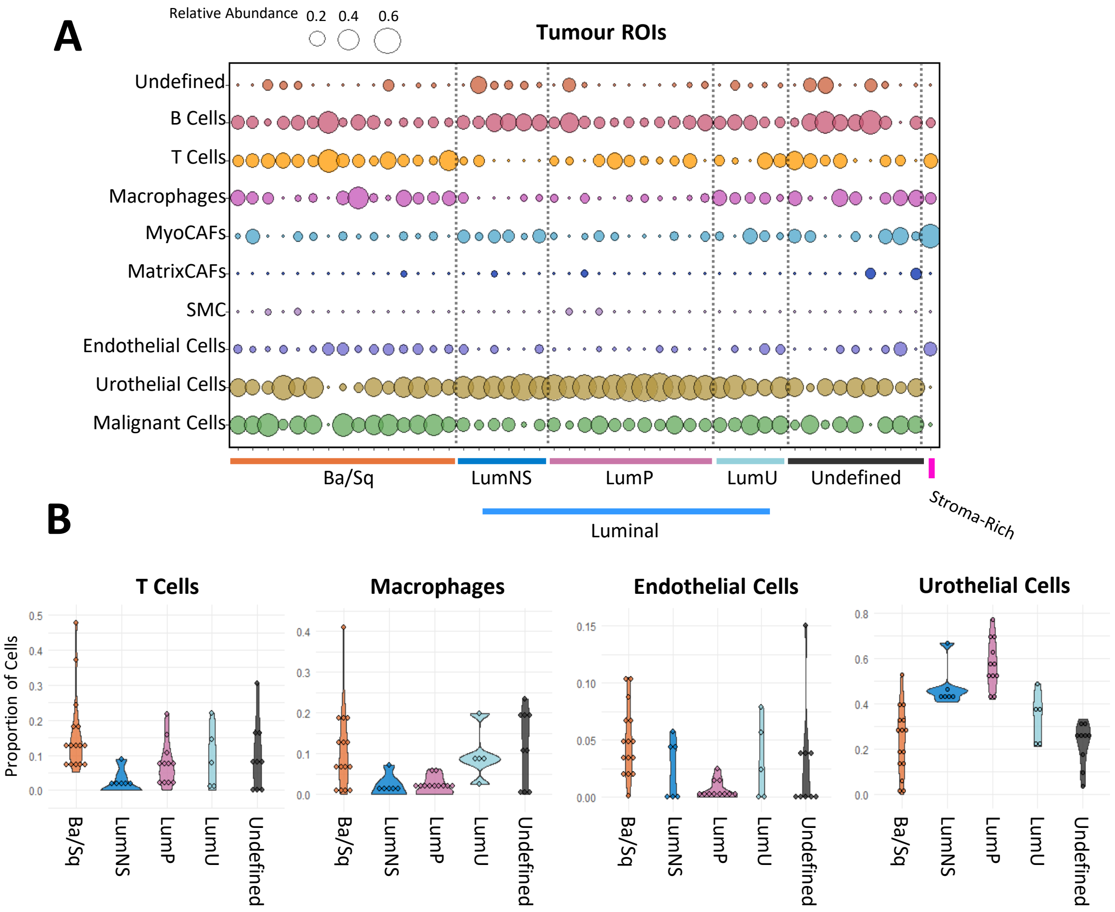

Supplementary Figure 3: Single-cell spatial deconvolution of tumour ROIs ordered by subtype. A) Bubble plot is a subset of Figure 2G that has been reordered to show differences in cell types by molecular subtypes. B) Violin plots showing differences in select cell types. Basal tumours were enriched in T Cells and macrophages relative to most luminal subtypes, while luminal subtypes were enriched in urothelial cells.

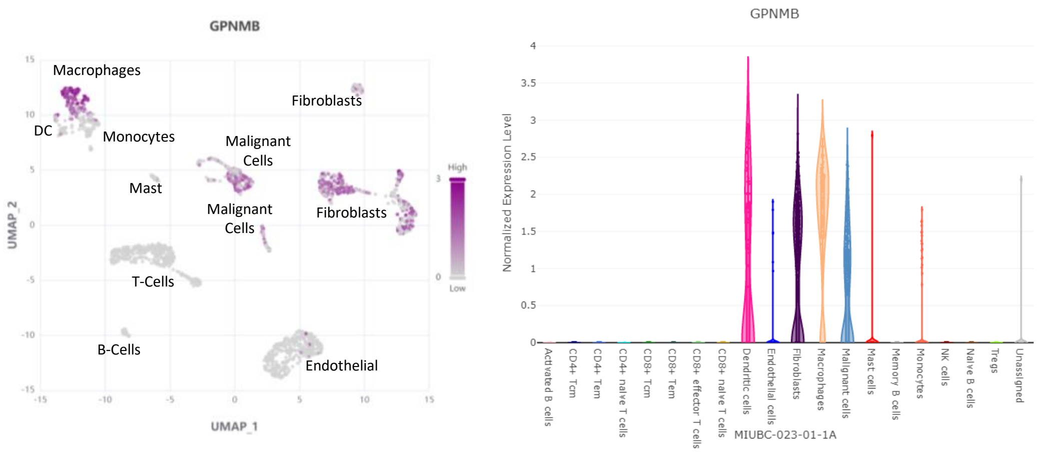

Supplementary Figure 4: Left) UMAP and (right) violin plots showing GPNMB expression in single-cell RNA-seq bladder cancer dataset by Lee et al (2020). GPNMB was highly expressed in Macrophages and subsets of fibroblasts and malignant cells.

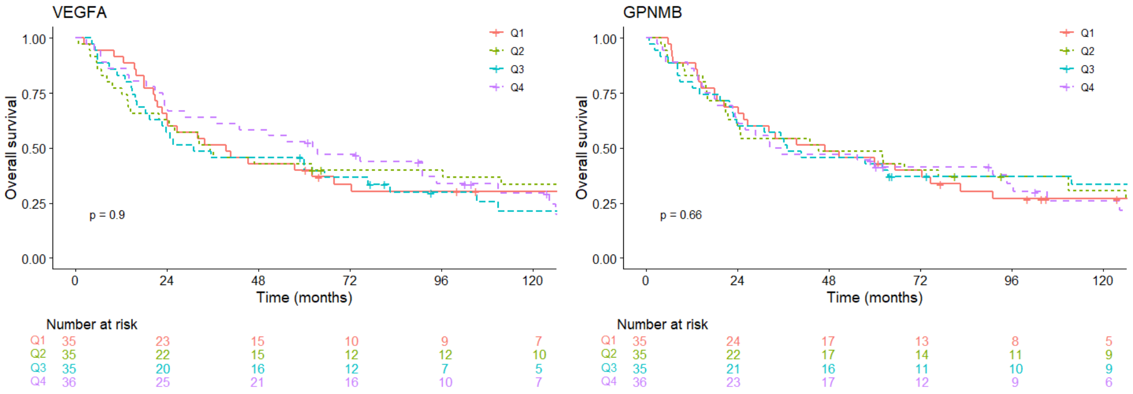

Supplementary Figure 5: Coxph survival analysis for (left) VEGFA and (right) GPNMB in a bulk RNA-sequencing dataset in the BCON cohort. There were no associations between GPNMB and VEGFA and overall survival seen in this dataset.

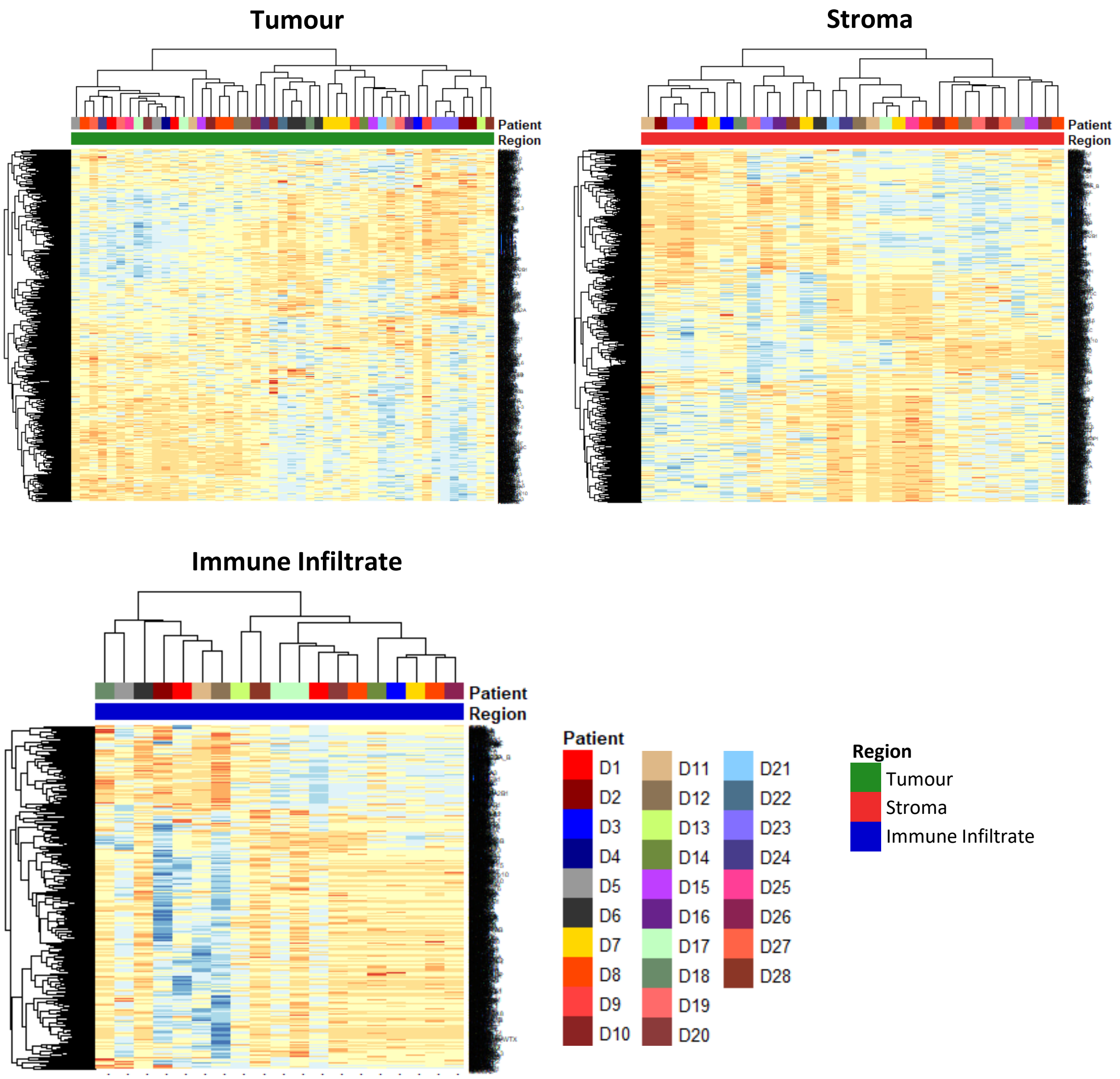

Supplementary Figure 6: Hierarchical clustering of top 1000 variable genes in tumour, stroma and immune infiltrate ROIs. Some ROIs taken from different cores from the same donor cluster together (5/11 for tumour, 1/7 for stroma/ 1/3 for immune infiltrate). However, many ROIs from the same donor do not immediately cluster together, suggesting high levels of intra-tumour heterogeneity.

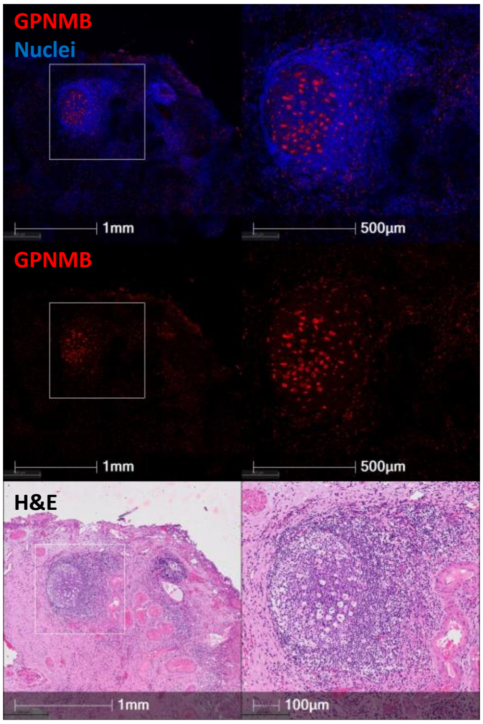

Supplementary Figure 7: Positive control staining for GPNMB in a lymph node showing strong positive staining in macrophages.
