# Supplementary material for: Digital spatial profiling of the microenvironment of muscle invasive bladder cancer
Source: Commun Biol. 2024 Jun 18;7:737. doi: 10.1038/s42003-024-06426-9 (PMC11189454; doi:10.1038/s42003-024-06426-9)
Supplement: Supplementary file 3 — Description of Additional Supplementary Files [file 42003_2024_6426_MOESM3_ESM.pdf]

Description of Additional Supplementary Files

File name: Supplementary Data

Description: Supplementary data consisting of raw, noramlised and log2 count data, annotation file, ROI region and subtype differentially expressed gene results, cell profile matrix for cell deconvolution, and R analysis script.
